# Clinical Performance of Viscous Glass Ionomer Cement in Posterior Cavities over Two Years

**DOI:** 10.1155/2009/781462

**Published:** 2010-02-22

**Authors:** Roland Frankenberger, Franklin Garcia-Godoy, Norbert Krämer

**Affiliations:** ^1^Department of Operative Dentistry and Endodontics, Medical Center for Dentistry, University Medical Center Giessen and Marburg, Georg-Voigt-Street 3, 35039 Marburg, Germany; ^2^College of Dentistry, University of Tennessee, 875 Union Avenue, Memphis, TN 38163, USA; ^3^The Forsyth Institute, 140 Fenway, Boston, MA 02115, USA; ^4^Department of Pediatric Dentistry, Medical Center for Dentistry, University Medical Center Giessen and Marburg, Schlangenzahl 14, 35392 Giessen, Germany

## Abstract

In this controlled prospective clinical study the highly viscous glass ionomer cement Ketac Molar was clinically assessed in Class I and Class II cavities. Forty-nine subjects (mean age 32.3 years) received 108 restorations placed by six operators in conventional Black I and II type cavities with undercuts after excavating primary lesions or after removing insufficient restorations. At baseline, and after 6, 12, and 24 months, restorations were assessed by two independent investigators according to modified USPHS codes and criteria. Impressions of the restorations were taken and epoxy replicas were made. Between the baseline and the 24-month recall, 51 representative samples were analyzed at 130 × magnification by use of a stereo light microscope (SLM). Recall rates were 83% after 6 months, 50% after 12 months, and 24% after 24 months. Failure rates after 24 months were 8% for Class I and 40% for Class II fillings, mainly due to bulk fracture at occlusally loaded areas (Kaplan Meier survival analysis). Significant changes over time were found for the criteria “surface roughness”, “marginal integrity”, “restoration integrity”, and “overall judgement” (*P* < .05; Friedman test). SLM analysis revealed statistically significant differences for the following criteria over time (baseline/6 months/12 months (in % of entire evaluable margin length); *P* < .05; Friedman 2-way ANOVA): perfect margin 37/19/11, negative step formation 26/49/57, gap formation 2/7/9, and overhang 24/11/8. Replicas exhibited mainly negative step formation as main finding due to apparently inferior wear resistance (*P* < .05). Gap formations were more frequently observed in Class II restorations than in Class I (12% versus 3% after 12 months; *P* < .05, Mann-Whitney-*U* test). The evaluated margin lengths were not statistically different (*P* > .05, Friedman 2-way ANOVA).

## 1. Introduction

Since the introduction of glass ionomer cements (GICs) by Wilson and Kent, many modifications of these materials have been performed over the years [1]. Classical GIC powder consists of silica, alumina, calcium fluoride as flux, cryolite, sodium fluoride, and/or aluminum phosphate. These raw materials are heated up to 1100–1500°C, resulting in fluoroaluminiumsilicate glass, which is milled to powder. The fluid phase is compolymerized acrylic and itaconic acid or maleic or tricarboxylic acid [2]. Water balance is fundamentally important for an optimum setting reaction, any difference during or shortly after the setting reaction severely decreases physical properties [3]. This particular problem was solved by introducing capsule materials [4–6]. 

In order to improve mechanical properties, manufacturers added silver (e.g., Ketac Silver, 3M Espe, Seefeld, Germany) or increased viscosity by reducing filler size (e.g., Ketac Molar, 3M Espe; Ionofil Molar, Voco, Cuxhaven, Germany; Fuji IX, GC, Tokyo, Japan) in order to achieve a certain packability [7–9]. 

GICs were characterized as fluoride releasing [10, 11], which also recently was found to be protective against biofilm challenge as restorative [12, 13] and as luting cement for metallic restorations [14]. On the other hand, clinical outcome was not automatically favorable when GIC was used as restorative material [15–17]. At least initially, the introduction of GIC was connected with hopes of being able to replace amalgam. Especially in Europe, this was an interesting aspect because amalgam was more and more disregarded during the 1990's [15–17] with many amalgam restorations having been replaced by GIC.

In primary molars, several studies showed that GIC is not recommendable for Class II cavities due to unacceptable high fracture rates; however, Class I cavities may be restored [18–24]. In the permanent dentition, prospective studies have been rarely published. However, retrospective trials repeatedly reported unsatisfactory clinical performance in Class II cavities [25]. Reviews indicated that the annual failure rate with GIC is estimated to be around 8% [15, 21]. 

Therefore, the aim of the present clinical trial was to prospectively calculate the potential of a highly viscous GIC for restoring posterior cavities in permanent molars. The null hypothesis was that there would be no difference between Classes I and II cavities regarding clinical outcome.

## 2. Materials and Methods

All patients were required to give written informed consent. The study was conducted according to EN 540 (Clinical investigation of medical devices for human subjects, European Committee for Standardization), and inspected by a local ethics committee. Al patients participated voluntarily. Patients selected for this study met the following criteria: (1) absence of pain from the tooth to be restored; (2) absence of any active periodontal and pulpal desease.

Fifty-five subjects (31 female, 24 male, mean age 33.0 years) received 108 GIC restorations. Twenty one restorations were placed in Class I and ninety four in Class II cavities. All restorations were made by four experienced dentists in a University dental clinic (31 bicuspids, 84 molars, 51 upper teeth, 64 lower teeth). Reasons for replacement were caries (*n* = 47), deficient restoration, that is, fracture or gap formation with exposed dentin (*n* = 68).

All restorations were inserted in permanent vital teeth without pain symptoms. For macromechanical retention, all cavities were made with undercuts. The cavities were cut using 1 mm wide coarse diamond burs under profuse water cooling (80 *μ*m diamond, Komet, Lemgo, Germany), and finished with a 25 *μ*m finishing diamond (Komet). Inner angles of the cavities were rounded and the margins were not bevelled. Prior to restorative treatment, the depth and width of the cavities was measured with a periodontal probe.

According to the manufacturer's recommendations, cavities were pretreated with Ketac Conditioner. Cavities were isolated with cotton rolls. Deep portions of the cavities (estimated remaining dentin thickness of <200 *μ*m) were covered with calcium hydroxide (Calxyl, OCO, Dirnstein, Germany). Ketac Molar Maxicap was mixed for 12 seconds in a RotoMix (3M Espe) apparatus. The GIC was applied into the cavity in one layer and adapted to the cavity walls with a plugger. The restorations were protected with Ketac Glaze (3M Espe) and light-cured for 20 seconds. 

After application of the glaze, rotary adjustment was performed at least 5 minutes later. Visible overhangs were removed with a scaler. Contacts in centric and eccentric occlusion were controlled with foils (Roeko, Langenau, Germany) and adjusted with finishing diamonds (Komet). Finally the restoration was shaped with silicon instruments (GC polishing set, GC Europe, Leuven, Belgium). 

At the initial recall (baseline), and after 6, 12, and 24 months, available restorations were assessed according to modified United States Public Health Service (USPHS) criteria by two independent investigators using mirrors, probes, bitewing radiographs, impressions (Dimension Penta and Garant, 3M Espe), and intraoral photographs. Recall assessments were not performed by the clinician who placed the restorations. Impressions were used to make epoxy replicas (Epoxy Die, Ivoclar Vivadent, Principality of Liechtenstein). 51 replicas were selected for stereo light microscopic (SLM) analysis, SLM replicas were assessed at 130-fold magnification under a stereo light microscope (SV 11, Zeiss, Jena, Germany) in combination with a 3 CCD color camera (Sony, Cologne, Germany) and a frame grabber (Matrox Meteor RGB, AVT Horn, Aalen, Germany). The KS 100 software (Jenoptik, Jena, Germany) was used for digitization and WinMes 2.0 software was used for margin analysis. Marginal integrity was expressed as percentage of the entire evaluable margin length. Marginal quality was classified according to the criteria “perfect margin”, “gap/irregularity”, “negative step”, “positive step”, “overhang”, and “not judgeable/artifact” [26]. 

Statistical appraisal was computed with SPSS for Windows XP 15.0 (SPSS Inc., Chicago, IL, USA). Statistical unit was one tooth, differences between groups were evaluated using the Mann-Whitney *U*-test, changes over time were calculated with the Friedman test (*P* = .05).

## 3. Results

**Figure 1:**
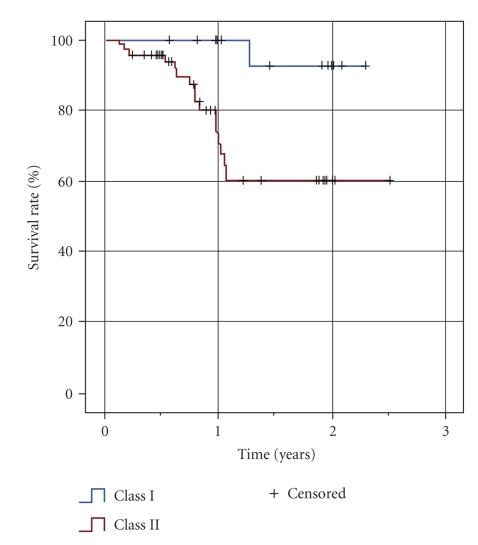
Kaplan-Meier survival curve.

Details about clinical outcome of the GIC restorations are displayed in Table 1. After 12 months, 54 restorations were evaluated, and after 24 months of clinical service, 26 restorations were available for recall assessments. The reasons for not qualifying for recall visits were missed recall (*n* = 52), prosthetic measures like crown preparations (*n* = 10), and other non-material-specific reasons like extraction (*n* = 2). This means a drop-out rate of 76% over the 24-month period. Failure rates after 24 months were 8% for Class I and 40% for Class II restorations (Kaplan-Meier survival curve; Figure 1). Seventeen Class II restorations had to be replaced due to material-specific reasons (bulk fracture *n* = 9; hypersensitivity *n* = 4; gap formation *n* = 3; tooth fracture *n* = 1; complete loss of the restoration *n* = 1; Figures 1, 3, and 4). 

**Figure 2:**
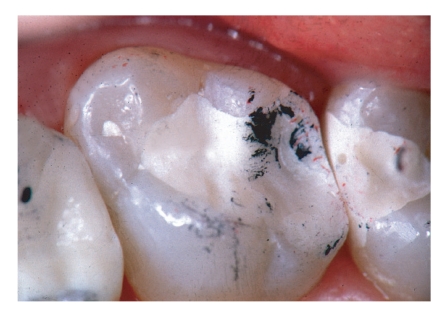
Ketac Molar restoration at baseline. The occlusal contact area was marked with Occlu-foil.

**Figure 3:**
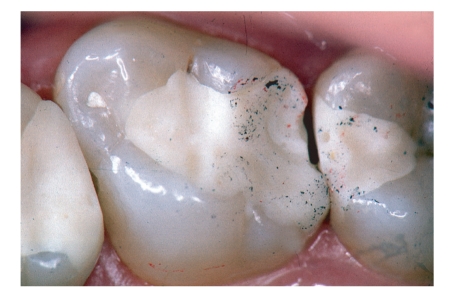
Ketac Molar restoration after 6 months. Chipping occured in the occlusal-proximal contact area.

**Figure 4:**
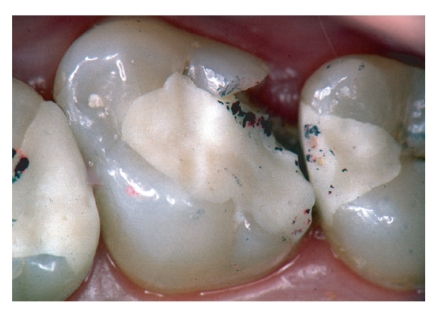
Ketac Molar restoration after 12 months. Half of the proximal box was lost.

Significant changes over time were found for the criteria “surface roughness”, “marginal integrity”, “restoration integrity”, and “overall judgement” (Friedman test; *P* < .05; Table 1). After 6 months, one “delta” resulted from a tooth fracture, and after 12 months, three “Charlie” ratings resulted from marginal fractures with lost proximal contact (Figures 2–4). One restoration suffered complete retention loss (“delta”; Table 1). 

**Figure 5:**
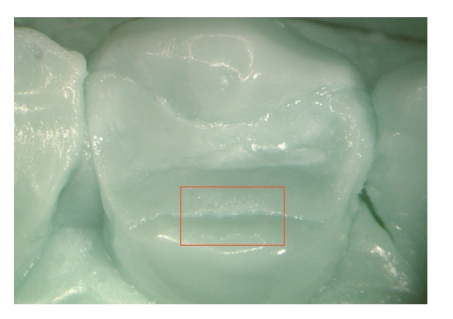
Epoxy replica of a Ketac Molar filling after 12 months. A distinct crevice is evident.

**Figure 6:**
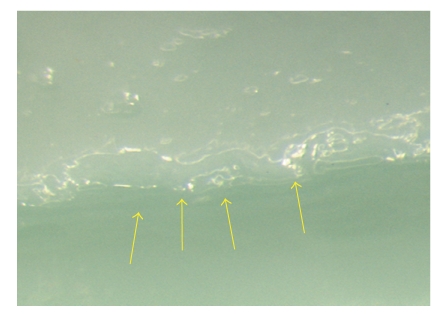
Magnification of the palatal aspect of Figure 5. No gap was detectable.

An overview of SLM observations is shown in Table 2. The results of the SLM marginal analysis revealed significant differences concerning the criteria “perfect margin”, changes in “positive step formation”, “negative step formation” (Figures 5 and 6), “overhang”, “marginal fracture”, and “gap formation” over time (Friedman test, *P* < .05; Table 2). Between cavity size and failure no correlation could be computed (Mann-Whitney *U*-test, *P* > .05; Table 3).

## 4. Discussion

The merits of GICs as restorative materials for both dentitions are clearly reflected by the literature in the field: GICs show fluoride release [7, 10, 11, 18] and consequently offer some potential to inhibit secondary caries [7, 10, 14, 27–30]. GICs were even discussed as pit and fissure sealing material with some protective aspects even after retention loss [8, 31, 32]. Furthermore, GIC is the ideal material for atraumatic restorative techniques [29, 33–36] and seems to reveal some advantages concerning long-term costs in routine dental restorative treatment because handling is quick and easy [9, 37, 38].

Although a variety of clinical trials with different kinds of GIC as permanent restorative materials was carried out in primary molars [18–24, 39, 40], not a single prospective clinical medium or long term trial is published about GIC in permanent molars. Retrospective studies primarily reported dissapointing outcomes when GIC was applied in average cavities [15, 21, 25] and slightly better results when minimum intervention cavities were restored with conventional GIC [41, 42]. This observation was not confirmed by the present study, because here no influence of cavity size on clinical outcome was found. Much more important was the occlusal contact situation (Figures 2–4) with a clear correlation of contact points on lateral ridges and bulk fractures exactly in these areas of special risk. And this is only true for Class II restorations with a higher load situation compared to Class I restorations where the brittle GIC material is always completely surrounded by enamel and dentin.

A retrospective study of Mjor doubted the previous assumption that GIC as permanent restorative material is really able to counteract secondary caries formation after a certain time of clinical service [43, 44]. The time span of the present investigation may be too short to contribute to this rather important question, however, several other shortcomings of GIC materials are clearly reflected here such as flexural fatigue behaviour.

When routine clinical long-term trials dealing with restorative materials or even more expensive indirect restorations are carried out accordingly, drop-out rates are normally within a low range. This enables the researcher to draw profound conclusions about clinical performance when a certain time of clinical service is over [15, 16]. From our experience it is obvious that the major disadvantage of prospective clinical trials dealing with cheapest and aesthetically compromised restorations such as GIC in permanent molars is drop out of patients. Of course, high drop-out numbers are potentially corroborating clinical conclusions and recommendations. This was the case in the present clinical investigation. All patients participated voluntarily, so they did not lose money or so when not showing up again. Nevertheless, there are some results with substantial impact on the overall judgement of the clinically investigated material.

Already after 12 months of clinical service, 34% of Class II GIC restorations had failed, meaning a 34% annual failure rate, which is five times higher than retrospective assumptions of cross-sectional studies exhibited [15, 21, 25]. Facing the circumstances of the reported rather high drop out in this study, we can conclude that at least one third of restorations had already failed after 12 months. We take the right to speculate that from the overall population of patients considerably more restorations failed during this 24 months period but were not available for recall. Even when defensively calculating the poor 24 months result, we still face a 20% annual failure rate which is still more than two times higher than reported in evaluations and reviews of a solely retrospective nature. Finally it has to be considered that the high drop out numbers definitely could bias the results.

Also marginal analyses under a SLM clearly show the inferior potential of GIC in Class II cavities being attributable to extensive wear and insufficient flexural strength as well as fatigue behaviour [45].

Altogether, the discussion about GICs is subclassified into two major aspects. On one hand, GICs offer fluoride release and easy handling properties, making them almost ideal for treatment of uncooperative children, covering of root caries, and performing ART projects. On the other hand, the moisture-sensitive setting reaction and the still way inferior flexural strength and flexural fatigue behaviour do not allow a recommendation of GICs for durable restorations of Class II cavities in permanent molars. Class I restorations exhibited a significantly better clinical outcome over the observation period of 24 months. Therefore, the null hypothesis had to be rejected because there was a distinct difference between Class I and Class II cavities.

## 5. Conclusions

Highly viscous GIC showed unacceptable high failure rates in Class II cavities, irrespective of cavity size. Annual failure rates of 20% are substantially higher than estimated from retrospective clinical trials. However, the high drop out observed in this study limits its significance.

## Figures and Tables

**Table 1:** Descriptive statistics for all Ketac Molar restorations.

Criterion	Baseline (*n* = 107)			6 months (*n* = 90)				12 months (*n* = 54)				24 months (*n* = 26)		
	Alpha	Bravo	Charlie	Alpha	Bravo	Charlie	Delta	Alpha	Bravo	Charlie	Delta	Alpha	Bravo	Charlie
	[%]			[%]				[%]				[%]		
Surface roughness	79	21		47	53			6	94				100	
Anatomic shape	74	26		73	27			63	35			73	27	
Marginal integrity	72	28		47	53			26	68	6		8	88	4
Integrity tooth	93	7		93	6		1	96	4			100		
Integrity filling	94	6		81	19			63	29	6	2	69	31	
Occlusion	87	13		91	9			86	14			83	17	
Proximal contact	69	31		80	20			80	10	10		73	27	
Change of sensitivity	100			100				100				100		
Hyper-sensitivity	100			98	2			100				100		
Overall judgement	73	27		49	50		1	35	54	11		27	69	4

**Table 2:** SLM margin analysis of all restorations.

	Baseline	6 months	12 months
	(*n* = 51)	(*n* = 48)	(*n* = 39)
Length [*μ*m]	15.6 × 10^3^	15.3 × 10^3^	17.2 × 10^3^
	(5.5 × 10^3^)	(5.4 × 10^3^)	(5.3 × 10^3^)
Criterion			
Perfect margin	36.0%	18.5%	10.9%
	(21.7)	(17.8)	(14.7)
Negative step	25.6%	49.3%	57.1%
Formation	(22.9)	(23.9)	(21.3)
Gap formation	2.1%	7.1%	9.8%
	(4.7)	(10.1)	(12.4)
Overhang	23.5%	11.3%	8.7%
	(20.1)	(13.1)	(9.8)
Positive step	6.1%	3.1%	1.7%
Formation	(9.3)	(8.1)	(5.8)
Marginal	0.0%	1.2%	1.7%
Fracture	(0.0)	(3.8)	(5.0)
Artifact	6.5%	9.3%	10.2
	(4.3)	(11.8)	(12.0)

**Table 3:** Cavity size related to survival of restorations after one year.

Measured margin length	Intact restorations		Fractured restorations		Level of significance
	N	mean [mm]	*n*	mean [mm]	(Mann-Whitney *U*-Test)
(1) Width at the isthmus					
(a) mesially	19	4.16	6	4.18	1.00
(b) distally	10	4.18	5	3.62	0.13
(2) Maximum width of proximal box					
(a) mesially	19	5.25	6	5.43	0.73
(b) distally	10	5.66	5	5.22	0.59
(3) Vestibular height at isthmus					
(a) mesially	14	2.83	6	3.73	0.13
(b) distally	8	3.39	4	4.30	0.11
(4) Height at isthmus					
(a) mesially	14	2.84	6	3.74	0.44
(b) distally	8	3.43	4	4.75	0.21
(5) Maximum cavity width Transversally	35	5.61	9	5.13	0.53
(6) Maximum cavity width mesio-distally	3	7.58	9	7.04	0.80
